# Microenvironment dependent gene expression signatures in reprogrammed human colon normal and cancer cell lines

**DOI:** 10.1186/s12885-018-4145-8

**Published:** 2018-02-27

**Authors:** Egle Strainiene, Mindaugas Binkis, Silvija Urnikyte, Vaidotas Stankevicius, Ausra Sasnauskiene, Gabrielis Kundrotas, Andrius Kazlauskas, Kestutis Suziedelis

**Affiliations:** 1 National Cancer Institute, Santariskiu 1, 08660 Vilnius, LT Lithuania; 2 Department of Chemistry and Bioengineering, Vilnius Gediminas Technical University, Vilnius, Lithuania; 3 Institute of Biosciences, Life Sciences Center, Vilnius University, Vilnius, Lithuania; 4 Institute of Biotechnology, Life Sciences Center, Vilnius University, Vilnius, Lithuania; 5 Department of Ophthalmology, Harvard Medical School, Schepens Eye Research Institute and Massachusetts Eye and Ear Infirmary, Boston, MA 02114 USA

**Keywords:** iPSC, Stem-like cancer cells, 3D cell culture, Genome wide analysis, KEGG analysis, Gene network analysis

## Abstract

**Background:**

Since the first evidence suggesting existence of stem-like cancer cells, the process of cells reprogramming to the stem cell state remains as an attractive tool for cancer stemness research. Current knowledge in the field of cancer stemness, indicates that the microenvironment is a fundamental regulator of cell behavior. With regard to this, we investigated the changes of genome wide gene expression in reprogrammed human colon normal epithelial CRL-1831 and colon carcinoma DLD1 cell lines grown under more physiologically relevant three-dimensional (3D) cell culture microenvironment compared to 2D monolayer.

**Methods:**

Whole genome gene expression changes were evaluated in both cell lines cultured under 3D conditions over a 2D monolayer by gene expression microarray analysis. To evaluate the biological significance of gene expression changes, we performed pathway enrichment analysis using the Kyoto Encyclopedia of Genes and Genomes (KEGG) database. Gene network analysis was used to study relationships between differentially expressed genes (DEGs) in functional categories by the GeneMANIA Cytoscape toolkit.

**Results:**

In total, we identified 3228 and 2654 differentially expressed genes (DEGs) for colon normal and cancer reprogrammed cell lines, respectively. Furthermore, the expression of 1097 genes was commonly regulated in both cell lines. KEGG enrichment analysis revealed that in total 129 and 101 pathways for iPSC-CRL-1831 and for CSC-DLD1, respectively, were enriched. Next, we grouped these pathways into three functional categories: cancer transformation/metastasis, cell interaction, and stemness. β-catenin (CTNNB1) was confirmed as a hub gene of all three functional categories.

**Conclusions:**

Our present findings suggest common pathways between reprogrammed human colon normal epithelium (iPSC-CRL-1831) and adenocarcinoma (CSC-DLD1) cells grown under 3D microenvironment. In addition, we demonstrated that pathways important for cancer transformation and tumor metastatic activity are altered both in normal and cancer stem-like cells during the transfer from 2D to 3D culture conditions. Thus, we indicate the potential of cell culture models enriched in normal and cancer stem-like cells for the identification of new therapeutic targets in cancer treatment.

**Electronic supplementary material:**

The online version of this article (10.1186/s12885-018-4145-8) contains supplementary material, which is available to authorized users.

## Background

A population of cancer cells within tumors that possess stem-like characteristics, have attracted a great deal of attention in recent years. [1–3]. This population of cancer cells is referred as cancer stem-like cells (CSCs), or often just as cancer stem cells. CSCs possess characteristics of tumor initiation capacity, long-term repopulation of cancer cells, long-term cell dormancy, genomic instability, and resistance to conventional chemo-radiation anticancer therapy. Despite the evidence for the existence of CSCs [4, 5], the origin of this cancer cell population remains unknown. The hypothetical origins of CSCs include (1) malignant transformation of tissue specific stem cells through accumulation of different mutations and epigenetic alterations; (2) cell de-differentiation involving epithelial - mesenchymal transition (EMT) [6]. In regard to tumor hierarchical concept, the CSCs population constitute only a small percentage within a tumor cell mass and its microenvironment [7, 8]. In addition, the tumor microenvironment (TME) plays a crucial role in promotion of CSCs proliferation and differentiation [9]. As indicated for many normal stem cells, CSCs are considered to reside in their surrounding specialized microenvironment, known as the stem cell niche. The CSCs niche is a part of the TME, which provides all the components of normal stem cells, and also possesses non-stem-like cancer cells. Therefore models with an enriched CSCs population could provide additional advantages to investigate TME dependent factors in cancer stemness [10].

The pluripotent state of somatic cells could be induced by the over-expression of four Yamanaka factors (Oct3/4, Sox2, Klf4 and c-Myc) [11]. Reprogramming of cancer cells into stem cell state, provides us with possibility to investigate processes linked to stemness and metastatic behavior from the very early phases of tumor development [12]. While some studies indicate that cancer cells reprogrammed to a pluripotent state in vitro lose their metastatic or tumorigenic potential in vivo [12, 13], Singovski et al. obtained totally opposite results using reprogrammed primary human colon cancer cells engrafted in mice [14].

3D cell cultures tend to mimic gene expression and cellular signaling patterns as well as phenotypic profiles of in vivo tissues more precisely compared to cell monolayers [15]. In addition, CSCs characteristics such as tumorigenicity, immunogenicity and genomic instability might be advantageous while designing a versatile model of cancer [16, 17]. Considering this, the application of reprogrammed cancer cells could provide a better model for the investigation of very early phases of tumor development and also support the development of therapeutic strategies that target CSCs [18].

Here we combined cellular reprogramming and three-dimensional (3D) cell culture microenvironment to reconstruct the signaling pathways and transcriptional networks which are activated in CSCs in a microenvironment dependent manner. In order to define the characteristics and fate of CSCs, we used induced pluripotent stem (iPSC) cells, generated from human normal colon epithelial cell line CRL-1831 and human colon carcinoma cell line DLD1 cells. Both reprogrammed cell lines were cultivated at (2D) monolayer and (3D) multicellular spheroid culture conditions and global gene expression differences in cells cultured at 3D versus 2D culture conditions were analyzed using DNA microarrays. Our results indicate that a total of 3228 and 2654 genes for colon normal and cancer reprogrammed cell lines, respectively, were significantly altered. Furthermore, 1097 genes were commonly regulated in both cell lines. KEGG enrichment analysis revealed that in total 129 and 101 pathways for iPSC-CRL-1831 and CSC-DLD1, respectively, were enriched. These KEGG pathways were grouped into cancer transformation/metastasis, cell interaction, and stemness functional categories, and β-catenin (CTNNB1) was confirmed as a hub gene of all three functional categories. Our results demonstrate that pathways important for cancer transformation and tumor metastatic activity are altered in both cell lines during the transition from 2D to 3D culture conditions, suggesting the advantage of our model for identification of potential therapeutic targets in cancer treatment.

## Methods

### Cell lines

The cell lines FHC (ATCC® CRL-1831), DLD1 (ATCC® CCL-221) and HEK293T (ATCC® CRL-3216) were obtained from the American Tissue Culture Collection (ATCC). FHC (CRL-1831) cells were cultured in DMEM/F12 medium (Gibco, Invitrogen) supplemented with 25 mM HEPES, 10 ng/ml cholera toxin (Sigma Aldrich, Germany), 0,005 mg/ml insulin, 0,005 mg/ml transferrin, 100 ng/ml hydrocortisone (all from Sigma Aldrich, Germany), and 10% FBS (Gibco, Invitrogen). DLD1 colon carcinoma cells were cultured in RPMI 1640 medium (Gibco, Invitrogen) supplemented with 10% FBS (Gibco, Invitrogen), 2 mM glutamine and 1% Pen-Strep. HEK293T packaging cells were cultivated in DMEM medium supplemented with 10% FBS (Gibco, Invitrogen), 2 mM glutamine and 1% Pen-Strep. Reprogrammed cell lines iPSC-CRL-1831, derived from human colon normal cell line CRL-1831, and CSC-DLD1, from human colon carcinoma cell line DLD1, respectively, were cultured in stem cell medium (SCM) in hESC qualified Matrigel (BD Biosciences) modified plates. SCM consisted of DMEM/F12 medium (Gibco, Invitrogen) supplemented with 1× MEM-NEAA, 1× Glutamax, 50 μM β-Mercaptoethanol, 10 ng/μl bFGF (all from ThermoFisher Scientific), and 20% knockout serum replacement (ThermoFisher Scientific) SCM medium was changed daily, and cells were passaged by manual dissociation with EDTA or dispase.

### Lentivirus production

Reprogrammed cell lines from human colon normal cell line CRL-1831, and human colon carcinoma cell line DLD1 were generated using the lentiviral transduction method. Lentiviral inducible expression vector TetO-FUW-OSKM (Addgene plasmid 20321) from Dr. Rudolf Jaenisch [19] and lentiviral vector FUdeltaGW-rtTA (Addgene plasmid 19780) from Dr. Konrad Hochedlinger [20] were used for lentivirus production. The packaging plasmid (pCMV-dR8.91) and the envelope plasmid (VSV-G/pMD2.G) were from the Dana-Farber Cancer Institute/Harvard Medical School (Boston, MA). Briefly, HEK293T lentivirus packaging cells were seeded at 3.8 × 10^6^ cells in 10 cm plates and cultured overnight in DMEM medium supplemented with 10% FBS and without antibiotics. To prepare OKSM (Oct3/4, Sox2, Klf4, c-Myc) or rtTA lentivirus, a mixture of packaging plasmid (0.9 μg), envelope plasmid (0.1 μg), OKSM vector (1 μg) (or rtTA) and Xtreme GENE Transfection Reagent (Roche Diagnostics, Germany) were mixed and incubated at room temperature for 30 min. The mixture of transfection was transferred to HEK293T cells that were approximately 70% confluent. After 18 h, the medium was replaced with a growth medium modified to contain 20% FBS, and the virus was harvested at 24 h after the medium switch. The viral harvest was repeated at 24-h intervals 3 times. The virus-containing media were pooled and centrifuged at 800×*g* for 5 min, and the supernatant was used to infect CRL-1831 and DLD1 cells.

### Generation of reprogrammed cell lines

CRL-1831 and DLD1 cells were plated at a density of 10,000 cells/cm^2^ and 4000 cells/cm^2^ in 6-well plates, respectively. The OKSM, rtTA lentivirus and 8 μg/ml polybrene (Sigma) was transferred to CRL-1831 and DLD1 cells. After 24 h, the medium was replaced with DMEM/F12 and/or RPMI growth medium for CRL-1831 and DLD1 cells, respectively. Transgene expression was induced by the addition 2 μg/ml Doxycycline (Sigma) 48 h postinfection and the medium was replaced with SCM. Successfully infected cells were selected on the basis of their morphology and reaction to alkaline phosphatase. The resulting cells were characterized by immunofluorescence microscopy using antibodies against Tra 1–60, Tra 1–81 and SSEA-4, respectively.

### 3D cell culture

To evaluate the changes in gene expression between 2D and 3D, iPSC-CRL-1831 and CSC-DLD1 cells were transferred to a multicellular spheroids culture. To avoid cell attachment to the well bottom, each well was precoated with 1% agarose in sterile PBS. Multicellular spheroids were formed from 600 iPSC-CRL-1831 and CSC-DLD1 cells, respectively, and then suspended in a 100 μL SCM medium without bFGF and plated in each well of 96 round-bottom well plates precoated with 1% agarose, and centrifuged at 500 x g for 20 min. Multicellular spheroids were photographed every second day with inverted optical microscope Eclipse TS100 and digital camera DS-Fi2 (Nikon, Japan). The multicellular spheroids size was evaluated using SpheroidSizer 1.0 [21]. Cells under 3D spheroid culture conditions were harvested at day seven for a total RNA extraction.

### EdU labeling and confocal immunofluorescence microscopy

EdU labeling was performed by using the Click-it® EdU Alexa Fluor® imaging kit (ThermoFisher Scientific). Briefly, EdU (5-ethynyl-2′-deoxyuridine) was added to the culture medium at a final concentration of 125 μM. After a 24 h incubation, spheroids were rinsed in PBS and fixed 4% paraformaldehyde (ROTH). EdU detection, based on a“click” reaction between EdU and the Alexa Fluor® 488 dye, was performed following the manufacturer’s instructions. Nuclei were counterstained with 5 μg/ml 4′6-diamino-2-phenylindole (DAPI) (Sigma Aldrich, St. Louis, MO, USA). Spheroids were sectioned using Leica CM1900 cryostat (section thickness ~ 50 μm). Next, cryosections were placed onto Superfrost microscope slides (Thermo Scientific, USA) and mounted with Mowiol (ROTH). Fluorescence images of spheroids cryosections were acquired with Leica TCS SP5 II Confocal microscope using 10× objective. Excitation wavelengths were 488 nm and 405 nm, respectively.

### Total RNA extraction

RNA was isolated from harvested cells using a GeneJET RNA Purification Kit (Thermo Fisher Scientific, Lithuania) according to the manufacturer’s instructions. The quantity and quality of RNA were evaluated using Nanodrop 2000c (ThermoFisher Scientific) and Bioanalyzer 2100 (Agilent Technologies, Santa Clara, CA, USA).

### Global gene-expression analysis

cRNA sample preparation, labeling and hybridization were performed according to the manufacturer’s instructions. Briefly, 0,5 μg of total RNA was used for cDNA synthesis and amplification using the Message™Amp aRNA kit (ThermoFisher Scientific). Then 825 ng of cRNA labeled with Cy3/Cy5 dyes using the Arcturus® TURBO labeling™ Cy™3/Cy™5 Kit (Applied Biosystems, Netherlands) were hybridized to Human 4x44k Oligonucleotide Microarrays (Agilent Technologies, USA) using the HS 400 hybridization station (Tecan, Switzerland). Microarray slides were scanned using a LS Reloaded scanner (Tecan, Switzerland) for microarray image analysis, and the data generated were further analyzed using ImaGene ver. 9.0 (BioDiscovery, USA) and GeneSpring GX ver. 11.0 (Agilent Technologies, USA) software. Loess normalization was performed to adjust microarray data for variation. Gene expression fold change above 1.5 (with *p*-value < 0.05) was defined as differentially expressed between two conditions. KEGG pathway enrichment analysis was performed using Webgestalt online source [22]. Network construction analysis using the GeneMANIA plug-in of Cytoscape was performed to predict the most related genes of our gene sets [23]. All of the microarray data was deposited in a GEO Dataset database, Accession number GSE93228, (http://www.ncbi.nlm.nih.gov/geo/query/acc.cgi?acc=GSE93228).

### Quantitative RT-PCR

A total of 500 ng of RNA was used for cDNA synthesis using the RevertAid RT Kit (Thermo Fisher Scientific, Lithuania) according to the manufacturer’s instructions. A quantitative real-time polymerase chain reaction (qPCR) was performed according to the manufacturer’s instructions. Briefly, for each reaction in a 48-well plate, 1 μl cDNA, 2 μl forward and reverse primer (2 μM), 10 μl Maxima SYBR Green qPCR MasterMix (2X) (Thermo Fisher Scientific) and 5 μl nuclease-free water was used. The relative change of gene expression was calculated by the ΔΔCt method with TATA-Box Binding Protein (TBP) as the gene used for sample normalization. Each data point is displayed as the mean ± standard deviation of three independent biological experiments. All primers were purchased from Biolegio (Netherlands) (Additional file 1: Table S2).

## Results

### 3D spheroid proliferation

**Fig. 1:**
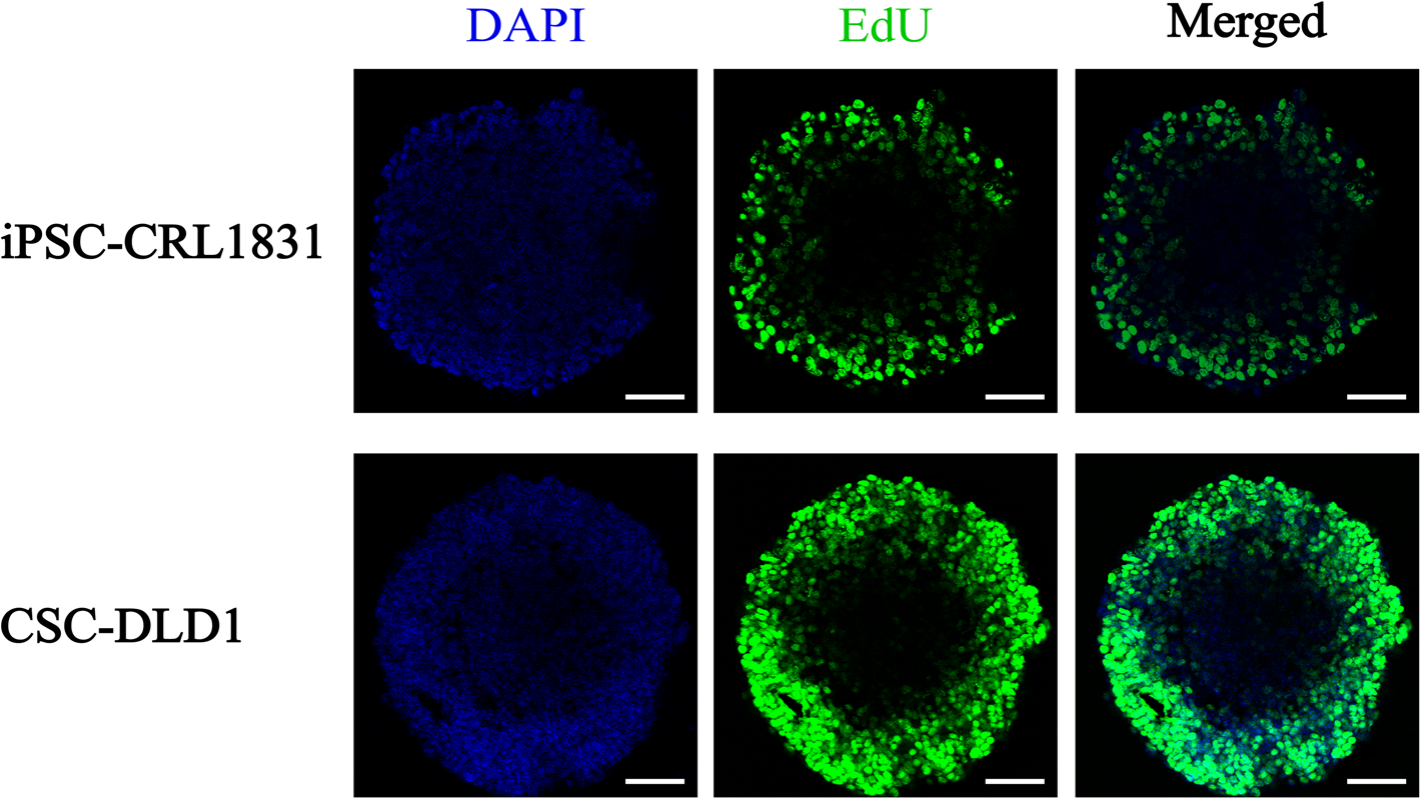
Cell proliferation regionalization in 3D spheroids. iPSC-CRL-1831 and CSC-DLD1 spheroids grown for seven days in the indicated conditions were incubated with EdU for 24 h prior to fixation and analysis. Representative confocal microscopy images of cryosections from spheroids. EdU detection was performed with Alexa FLuor® 488 (green). Nuclei were counterstained with DAPI (blue). Scale bar, 100 μm

Multicellular spheroids from reprogrammed CRL-1831 and DLD1 (induced pluripotent stem cells (iPSC-CRL-1831) and cancer stem-like cells (CSC-DLD1)) were prepared by cell-aggregation and cultured for seven days in a low- attachment 96 well plate. In order to evaluate the proportion of proliferating cells, we incubated spheroids with EdU for 24 h. Furthermore, to inspect precisely layer of the dividing cells or cells that have undergone replication, we performed spheroids staining and cryosectioning followed by confocal microscopy. As shown in Fig. 1, we obtained a clear regionalization of EdU incorporation restricted to the outermost layers of spheroids. This indicated that at day seven only cells located in the outer layer of the spheroids had undergone S-phase replication, while the remaining cells were at the dormant state.

### Gene expression pattern

**Fig. 2:**
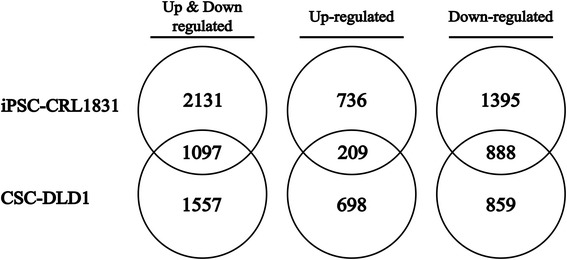
Venn diagrams showing the number of deregulated, upregulated and down-regulated differentially expressed genes (fold change of at least 1.5 and *p* < 0.05) in iPSC-CRL-1831 and CSC-DLD1 cells following seven days growth under 3D spheroid culture conditions

To evaluate the effect of 3D culture conditions compared to 2D in both reprogrammed cell lines, genome wide gene expression analysis was performed using Human Gene Expression (v2) 4x44k Oligonucleotide Microarrays. Among differentially expressed genes, a total of 3228 and 2654 genes (Table 1) for iPSC-CRL-1831 and CSC-DLD1, respectively, were detected to be significantly altered (*p* < 0.05; FC > 1.5). Most of the DEGs while in 3D conditions, revealed a tendency to be down-regulated in both cell lines. Furthermore, iPSC-CRL-1831 and CSC-DLD1 showed 1097 commonly regulated genes – among those, 209 were up-regulated while 888 tended to be down-regulated (Fig. 2).

**Table 1:** Number of differentially expressed genes in iPSC-CRL-1831 and CSC-DLD1

Cell line	Total (*p* < 0.05; FC > 1.5)	Up-regulated	Down-regulated
iPSC-CRL-1831	3228	945	2283
CSC-DLD1	2654	907	1747

### KEGG pathway enrichment analysis

To gain more insight into the biological significance caused by the 3D environment, we performed enrichment analysis using the KEGG database. The analysis revealed that in total 129 and 101 KEGG pathways for iPSC-CRL-1831 and CSC-DLD1, respectively, were enriched (*p* < 0.05). For further analysis, we grouped these pathways into 3 functional categories: cancer transformation/metastasis, cell interaction, and stemness (Table 2 and Additional file 1: Table S1), thought to be involved in the development of primary and metastatic tumors in vivo. Most of the identified pathways were altered in both cell lines. Our results revealed a high number of genes enriched in the cell interaction category involving genes associated with cell-cell and cell-ECM interaction processes. For the iPSC-CRL-1831 cell line, the most enriched set of genes in this category was adherens junction (*p* = 5.11 × 10^− 6^) while for CSC-DLD1 – focal adhesion (*p* = 1 × 10^− 4^). In addition, the Wnt signaling pathway for both iPSC-CRL-1831 and CSC-DLD1 cell lines (*p* = 0.003 and *p* = 8.54 × 10^− 5^, respectively) was the most enriched pathway in the stemness category. Furthermore, the most significantly enriched subset of genes in iPSC-CRL-1831 belonged to the pathways in cancer (*p* = 1.36 × 10^− 9^) and contained the highest number of DEGs – 55. Whereas, CSC-DLD1 showed the highest enrichment in the MAPK signaling pathway (*p* = 3.80 × 10^− 6^) with 36 DEGs. Both of these subsets were assigned to the cancer transformation/metastasis functional category. Moreover, nearly half of the genes in categories mentioned above were common for both reprogrammed cell lines, and thus indicated similar changes after the transfer from the 2D to 3D system.

**Table 2:** Common dysregulated pathways identified by KEGG database in iPSC-CRL-1831 and CSC-DLD1

Functional categories	iPSC-CRL-1831		CSC-DLD1		Common genes
	*p* value	Genes	*p* value	Genes	
Cell interaction					
Adherens junction	5.11e-06	18	0.0014	12	6
Tight junction	0.0001	22	0.0157	14	9
Regulation of actin cytoskeleton	0.0013	27	0.0006	25	10
Cell adhesion molecules (CAMs)	0.0040	18	0.0301	13	10
Focal adhesion	0.0043	24	0.0001	26	10
Gap junction	–	–	0.0273	10	–
ECM-receptor interaction	–	–	0.0394	9	–
Stemness					
Wnt signaling pathway	0.0003	23	8.54e-05	22	10
TGF-β signaling pathway	0.0110	12	0.0381	9	5
Hedgehog signaling pathway	0.0130	9	0.0156	8	4
Notch signaling pathway	0.0365	7	0.0172	7	2
Cancer transformation/metastasis					
Pathways in cancer	1.36e-09	55	4.18e-05	38	16
P53 signaling pathway	2.65e-05	16	0.0002	13	7
Colorectal cancer	0.0003	14	0.0103	9	3
VEGF signaling pathway	0.0009	14	–	–	–
MAPK signaling pathway	0.0070	29	3.80e-06	36	10
mTOR signaling pathway	0.0088	9	–	–	–
Apoptosis	0.0297	11	0.0113	11	5

Common genes: number of common genes included in both cell lines

### Heat map analysis

**Fig. 3:**
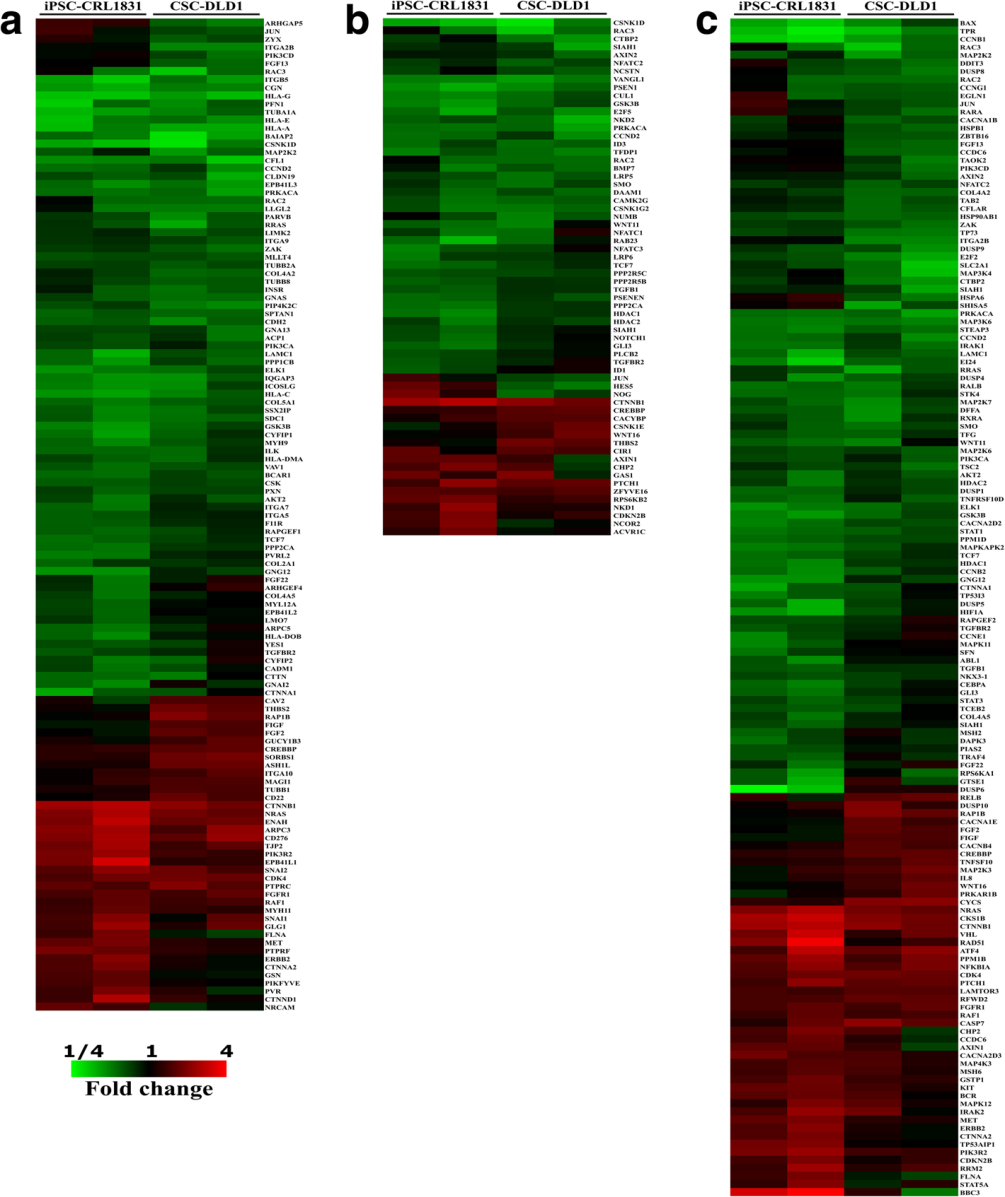
Heat map analysis of KEGG database selected categories. Heat maps representing the expression profile for cell interaction (**a**), stemness (**b**), cancer transformation/metastases (**c**) genes following seven days of iPSC-CRL-1831 and CSC-DLD1 cell growth under 3D cell culture conditions compared to 2D monolayer. Red and green indicate an increase and decrease of gene expression, respectively

Genes corresponding to the mentioned categories for both cell lines were represented in the form of heat maps (Fig. 3). Surprisingly, the gene expression signatures between both lines remain very similar under 3D culture conditions, with the majority of the genes being down-regulated. Figure 3a encompasses differentially expressed genes from the cell interaction category with a total number of 123 DEGs. This category is comprised of genes belonging to the adherens junction, tight junction, regulation of actin cytoskeleton, cell adhesion molecules (CAMs), focal adhesion, and ECM-receptor interaction subsets. Several groups of genes can be distinguished in this category. Most of the integrins including ITGA5, ITGA7, ITGA9, ITGA10, ITGA2B, ITGB5 are down-regulated. In addition to this, a group of human leukocyte antigens including HLA-A/C/E/G, HLA-DMA and HLA-DOB are down-regulated in both cell lines under 3D cell culture conditions. Also, the heat map analysis depicted a group of tubulins (TUBA1A, TUBB2A, TUBB1 and TUBB8) enriched in CSC-DLD1.

Figure 3b depicts DEGs from the stemness category which includes Wnt, TGF-β, Hedgehog and Notch signaling pathways. There are 63 genes in this category. Expression pattern of most of these genes is very similar between both lines, except for a few – JUN, HES5, NOG and NCOR2 which indicated a clearly opposite regulation in iPSC-CRL-1831 and CSC-DLD1 cell lines under 3D cell culture conditions.

Last, Fig. 3c represent a total of 153 DEGs grouped in the cancer transformation/metastases category including pathways in cancer, p53, colon cancer, VEGF, MAPK, mTOR signaling pathways and apoptosis. We observed enrichment of this category by a group of the Ras superfamily coding genes (NRAS, RRAS, RAC2, RAC3, RALB, RAP1B). In addition, a group of dual specificity phosphatases including DUSP1, DUSP4, DUSP5, DUSP6, DUSP8, DUSP9, DUSP10 and most of those are down-regulated reside in this category, as well. Also, a large group of mitogen-activated kinases MAP2K2, MAP2K3, MAP2K6, MAP2K7, MAP3K4, MAP3K6, MAP4K3, MAPK11, MAPK12 and MAPK-activated protein kinase MAPKAPK2 show a different expression in this category.

### Gene network analysis

**Fig. 4:**
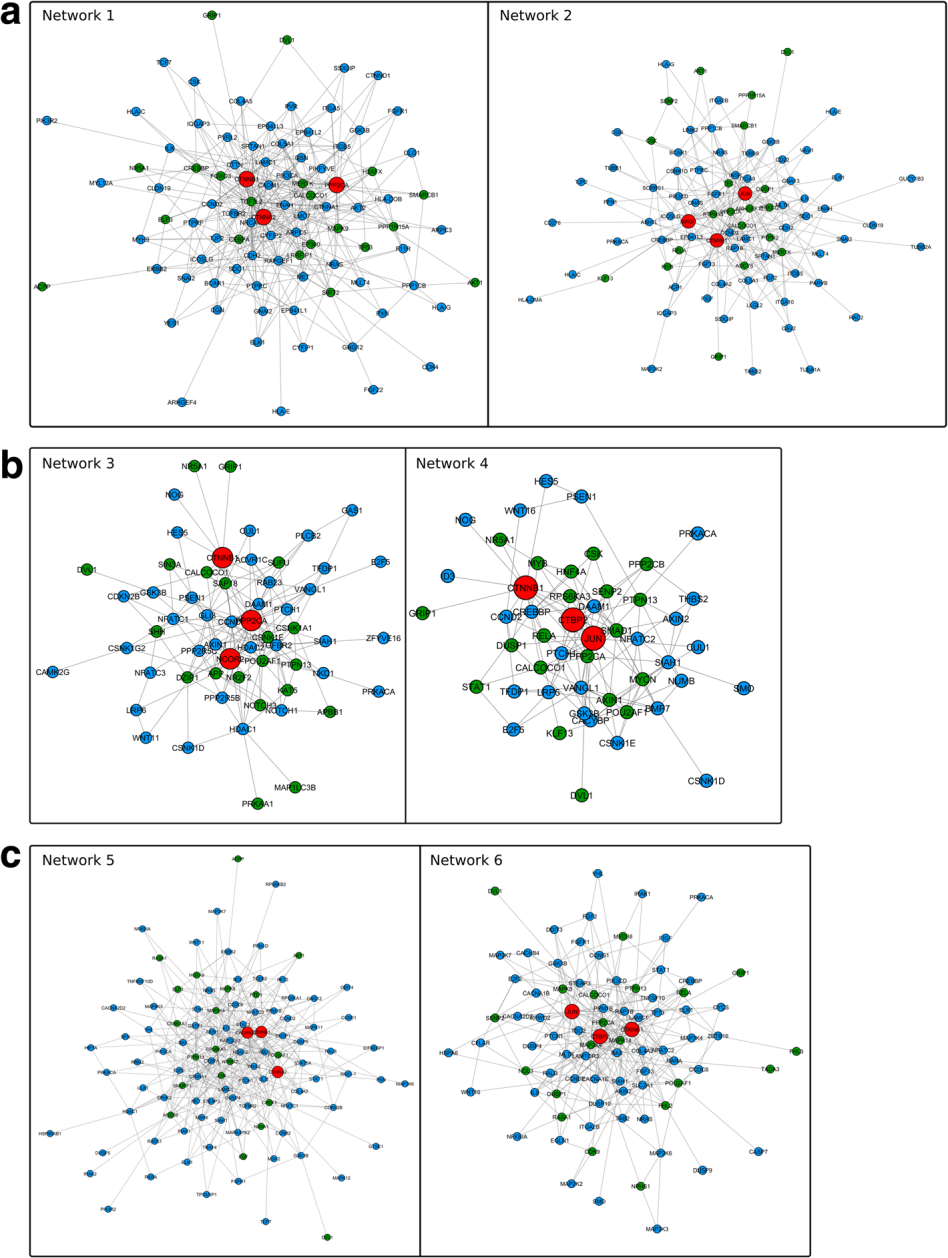
The interaction network of genes sets (cluster obtained by ClusterViz from GeneMANIA and visualized in Cytoscape). The interaction networks of significantly enriched and predicted genes for cell interaction (**a**), stemness (**b**), cancer transformation/metastases (**c**) categories. Networks 1, 3, 5 and networks 2, 4, 6 represent different categories for iPSC-CRL-1831 and CSC-DLD1, respectively. Nodes in blue are selected genes, nodes in green are GeneMania predicted genes, nodes in red represent hub genes. Totally, we identified 9 hub genes: CTNNB1, CTNNA1, CTNNA2 - catenin (cadherin-associated protein), beta1 and/or alpha1 and/or alpha2; PPP2CA - protein phosphatase 2, catalytic subunit, alpha isozymer; JUN - jun proto-oncogene; MAGI1 - membrane associated guanylate kinase, WW and PDZ domain containing 1; NCOR2 - nuclear receptor corepressor 2; CTBP2 - C-terminal binding protein 2; CACNA2D3 - calcium channel, voltage-dependent, alpha 2/delta subunit 3

For further analysis we performed a network construction of genes belonging to the three categories mentioned earlier in order to identify hub genes involved in the processes associated with cancer transformation/metastases, stemness, and cell interaction in iPSC-CRL-1831 and CSC-DLD1 cells grown under 3D cell culture conditions. Genes with the highest node degree were considered as hub genes of the network. Network 1 and Network 2 (Fig. 4a), represent the cell interaction category for iPSC-CRL-1831 and CSC-DLD1, respectively. Network 1 contains 71 significantly enriched genes and 20 predicted, while Network 2 consists of 64 identified DEGs and 20 predicted genes. CTNNB1 with 27 node degrees, CTNNA1 with 26 node degrees and PPP2CA with 20 node degrees were identified as hub genes in Network 1, whereas hub genes for Network 2 were CTNNB1–24, JUN – 20 and MAGI1–18 node degrees.

Network 3 and Network 4 (Fig. 4b) consist of genes from the stemness category for iPSC-CRL-1831 and CSC-DLD1, accordingly. Network 3 is composed of 38 identified DEGs and 20 predicted genes, while Network 4 contains 29 DEGs and 20 predicted genes as well. PPP2CA, NCOR2 and CTNNB1 were considered as hub genes in Network 3 having 18, 13 and 12 nodes, respectively. In Network 4 CTNNB1, JUN and CTBP2 were identified as hub genes having 15, 14 and 11 nodes, respectively.

For the cancer transformation/metastasis category Network 5 and Network 6 were constructed (Fig. 4c). Network 5 reflecting genes from iPSC-CRL-1831 contained 86 identified DEGs and 20 predicted genes. Meanwhile, Network 6 with genes from the CSC-DLD1 cell line consisted of 64 genes from the cell interaction category and 20 predicted genes identified by network construction analysis. CTNNA2 with 25 node degrees, CTNNB1 with 23 and CACNA2D3 with 20 were identified as hub genes for Network 5. CTNNB1 with 25 node degrees, JUN with 22 and CTBP2 with 18 – as hub genes for Network 6. CTNNB1 was identified as the main hub gene assigned to all three categories with a top node degree in CSC-DLD1, while in iPSC-CRL-1831 it was among the top three hub genes, indicating its essential role.

### Microarray gene expression data validation

**Fig. 5:**
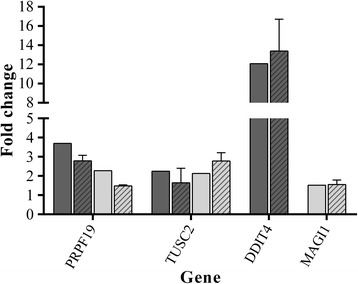
Validation of microarray gene expression data by qPCR using selected genes. The fold change of selected genes were normalized to TBP in RT-qPCR (expression in iPSC-CRL-1831 (dark bars) and CSC-DLD1 (light bars) cells grown under 3D cell culture conditions compared to 2D monolayer). Each bar represents the mean and standard deviations of three independently performed experiments for RT-qPCR. Bars with no pattern represent fold change of the microarray experiment (where *p*-value < 0.05), while bars with pattern - RT-qPCR

To validate selected data from microarray experiments, we used qRT-PCR for gene expression analysis. Thus, we took genes PRPF19 and TUSC2 that were common for both cell lines and DDIT4 and MAGI1 that were unique for iPSC-CRL-1831 and CSC-DLD1 cells, respectively. The fold change ratios of selected genes are displayed in Fig. 5. PRPF19 and TUSC2 represent genes that show increased expression in both cell lines grown under 3D versus 2D conditions. DDIT4 induction was only observed in iPSC-CRL-1831 and MAGI1 – in CSC-DLD1. Overall, a correlation between the microarray and qPCR results was observed.

## Discussion

Specialized niches of TME play crucial roles in defining the behavior of tumor cells, including CSCs [9]. Three-dimensional (3D) cell culture systems are introduced in cancer research as gene expression and cellular signaling patterns as well as phenotypic characteristics of 3D cultured cells tend to mimic profiles of in vivo tissues more precisely compared to monolayer cell cultures [15]. Considering this, 3D culturing of reprogrammed normal colon and carcinoma cells may provide an appropriate model to investigate the maintenance of cancer stemness.

In this study, we compared genome-wide expression profiles of reprogrammed human colon normal CRL-1831 and carcinoma DLD1 cells grown in more physiologically relevant three-dimensional (3D) versus traditional monolayer (2D) cell cultures. Microarray results revealed changes in 3228 and 2654 DEGs for both reprogrammed cell lines – CRL-1831 and DLD1, respectively. Moreover, these data demonstrated that CRL-1831 – colon normal and DLD1 – colon cancer cell lines share many common pathways after being reprogrammed and cultured under 3D conditions. In total, we identified 16 common altered pathways in both cell lines and attributed these pathways into the three functional groups: cell interaction, stemness, and cancer transformation/metastasis. The highest enrichment score for the iPSC-CRL-1831 cell line was ascribed to adherens junction (R = 3.88) (the second highest in CSC-DLD1 with R = 3.24), while in the CSC-DLD1 cell line – to the p53 signaling pathway (R = 3.77) (the second highest in iPSC-CRL-1831 with R = 3.71), suggesting the significance of cell interaction and p53 signaling in stem-like cells under the physiologically relevant microenvironment.

We identified several groups of DEGs in both reprogrammed cell lines that could be associated to the cancer cell resistance to a particular type of cancer treatment. One of the group – integrins, was assigned to the cell interaction functional category, while another group – MAP kinases, to the cancer transformation/metastasis category. Specifically, the integrin group consisted of six differentially regulated genes (ITGA5, ITGA7, ITGA9, ITGA10, ITGA2B, and ITGB5). In accord to our observation, changes in an integrin expression under 3D cell culture conditions resulted in an altered proliferation and apoptosis, and therefore increased cellular resistance to radiation and chemotherapy [9, 24]. Another group – MAP kinases consisted of nine differentially expressed genes (MAP2K2, MAP2K3, MAP2K6, MAP2K7, MAP3K4, MAP3K6, MAP4K3, MAPK11, and MAPK12), indicated changes in the MAPK signaling pathway as well. Gangadhara et al. demonstrated that cells cultured in 3D conditions promote a shift from PI3K/AKT to MAPK resulting in the reduced sensitivity to therapeutic agents as well [25], as in the studies mentioned above. Related to this, the regulation of the genes coding Ras superfamily was also altered. Previous studies have pointed out small GTPases as targets of the MAPK signaling pathway, in promoting cancer transformation processes [26]. Interestingly, these changes were observed in both cell lines suggesting the application of normal cell lines in cancer remodeling research.

We also discovered an additional family of deregulated genes belonging to the cell interaction functional category. However the expression of the tubulins gene family, including TUBA1A, TUBB2A, TUBB1 and TUBB8, changed only in the CSC-DLD1 cell line. Most of these genes were down-regulated. Whereas other studies demonstrated that the microtubules are involved in regulation of p53 activity by its translocation to the nucleus in a complex with heat shock protein 90 (HSP90), which provides stability for the p53 protein [27–29]. Microarray results revealed a down-regulation of genes encoding HSP90 in both cell lines as well, fortifying the supposition that the changes in p53 regulation are influenced by 3D cell culture conditions. These changes in regulation of the p53 signaling pathway might be as well related to the maintenance of the stemness state under the specific microenvironment. This probably leads to the maintenance of the stem-like phenotype, together increasing the precedent for the changes in cell death regulation and proliferation promotion as discussed above.

Furthermore, a decrease in the expression level of a group of human leukocyte antigen (HLA) encoding genes was observed in both reprogrammed cell lines. These findings are consistent with the Pick et al. study where he also reports the down-regulation of HLA genes of reprogrammed cells and points out that there exists a positive correlation between HLA and nuclear factor kappa B 1 (NFκB1) expression [30]. Since HLA genes are responsible for major histocompatibility complex (MHC) protein expression, this could also serve positively in modulation of cancer cell mechanisms involved in evading immune response.

Although, we were not able to identify any changes in NFκBI regulation, but the expression of NFKBIA increased. Carter et al. study demonstrated that NFKBIA gene, which encodes IκBα – a negative regulator of NF-κB and p53 signaling – suppresses apoptosis [31]. So, this might indicate the ability of iPSC-CRL-1831 and CSC-DLD1 cells to adapt to 3D environment, thereby increasing self-survival capabilities.

In addition, despite that the majority of stemness-related genes were down-regulated, a few that are important for proliferation and self-renewal were up-regulated. Among those, we detected that the expression of CREBBP and CTNNB1 genes increased. Lenz and Kahn demonstrated that the interaction between these two gene products – CBP (*cyclic AMP response element binding protein (CREB) binding protein,*) and β-catenin – initiates gene transcription, that is responsible for the maintenance of proliferative and potency (pluri- or multipotent) states [32]. This might be one of the factors promoting the stemness status in our experimental system.

The importance of β-catenin (CTNNB1) was also confirmed by the network analysis indicating it as a hub gene in all three functional categories with the highest node degree in the CSC-DLD1 cell line, while in the iPSC-CRL-1831 it was among the top three hub genes. Thus, the expression of CTNNB1 significantly increased (2.54 and 1.97, respectively) in both reprogrammed cell lines under 3D culture conditions. In addition, the up-regulation of the transcriptional activator CTNNB1 in the cell might be determined by the disturbance of a protein complex called ‘destruction box’. This complex was inactivated through GSK-3β (*glycogen synthase kinase 3 beta*) gene down-regulation, which is one of the components in the β – catenin disruption complex [33, 34]. Furthermore, the tumor suppressor p53 was implicated in the down-regulation of β-catenin expression and/or activity by Siah-1 dependent degradation [33], so the opposite regulation of β-catenin observed in our microarray data gives one more reason to consider down-regulation of the p53 signaling pathway in iPSC-CRL-1831 and CSC-DLD1 cell lines. Additionally, the expression of SIAH1 decreased in both cell lines. Also, the results above are consistent with the role of p53 in up-regulating the BAX gene which is involved in p53-mediated apoptosis [35]. Microarray results indicated this gene to be significantly down-regulated in both – iPSC-CRL-1831 and CSC-DLD1 cell lines (2.72 and 1.72 fold change, respectively) which suggests promotion of apoptosis suppression and activation of other cell death related mechanisms. The study conducted by Lee et al. also revealed that a decrease in BAX gene expression is associated with promotion of metastatic features in colon cancer [36]. Interestingly, this change of expression was common for both reprogrammed cell lines – colon normal and carcinoma.

In addition to our results, we also identified two signaling pathways from cancer transformation/metastasis functional category specific only to the iPSC-CRL-1831 cell line. These pathways are VEGF and mTOR. Since changes in gene regulation belonging to the mentioned pathways suggest promotion of proliferation and inhibition of apoptosis, it might explain tendency of iPSC-CRL-1831 to display properties typical for cancer cells after differentiating from the pluripotent state under 3D cell culture conditions. Observations reported by Souček et al. where they showed that the fetal human cell line FHC (CRL-1831) from normal fetal colonic mucosa exhibits a tumorigenic phenotype [37], supports this suggestion as well.

## Conclusion

Our study indicated that reprogrammed human colon normal and colon carcinoma cell lines share a common profile of gene expression under more physiologically relevant 3D spheroid versus 2D monolayer cell culture conditions. The result of the KEGG pathway enrichment analysis demonstrated that downstream signaling events of the p53 pathway might lead to an inhibition of cell cycle arrest, cellular senescence, and apoptosis. Finally, our findings demonstrate that pathways important for cancer transformation and tumor metastatic activity are altered during the transition from 2D to 3D culture conditions both in normal and cancer stem-like cells. Thus, we indicate the potential of cell culture models enriched in normal and cancer stem-like cells for the identification of new therapeutic targets in cancer treatment.

## Additional file

Additional file 1 **Table S1.** Common dysregulated pathways identified by KEGG database in iPSC-CRL-1831 and CSC-DLD1. **Table S2.** Primer sequences used in RT-qPCR. (DOCX 42 kb)

## Abbreviations

2D: two-dimensional
3D: three-dimensional
CSC: Cancer stem-like cells
DEGs: Differentially expressed genes
iPSC: induced pluripotent stem cell
