# Supplementary material for: Microenvironment dependent gene expression signatures in reprogrammed human colon normal and cancer cell lines
Source: BMC Cancer. 2018 Feb 27;18:222. doi: 10.1186/s12885-018-4145-8 (PMC5827990; doi:10.1186/s12885-018-4145-8)
Supplement: Supplementary file 1 — Table S1. Common dysregulated pathways identified by KEGG database in iPSC-CRL-1831 and CSC-DLD1. Table S2. Primer sequences used in RT-qPCR. (DOCX 42 kb) [file 12885_2018_4145_MOESM1_ESM.docx]

**Additional file 1:**

**Table S1. Common dysregulated pathways identified by KEGG database in iPSC-CRL1831 and CSC-DLD1.**

| Functional categories | iPSC-CRL1831 | | | CSC-DLD1 | | | |
| --- | --- | --- | --- | --- | --- | --- | --- |
|  | ***p* value** | **Genes** | | ***p* value** | | **Genes** | |
|  |  | **Up-regulated** | **Down-regulated** |  |  | **Up-regulated** | **Down-regulated** |
| Cancer transformation/metastasis | | | | | | | |
| Pathways in cancer | 1.36e-09 | CDKN2B, RAF1, ERBB2, GSTP1, CTNNA2, CTNNB1, PIK3R2, MET, KIT, MSH6, BCR, NRAS, VHL, CDK4, PTCH1, FGFR1, CCDC6, RAD51, AXIN1, NFKBIA, STAT5A | NKX3-1, CTNNA1, RXRA, COL4A5, CEBPA, TGFBR2, CCNE1, ABL1, GLI3, RALB, MSH2, PIK3CA, BAX, TFG, STAT3, TGFB1, LAMC1, TCEB2, HSP90AB1, FGF22, AKT2, WNT11, TCF7, DAPK3, TPR, HIF1A, STAT1, PIAS2, GSK3B, HDAC1, TRAF4, E2F2, HDAC2, STK4 | | 4.18e-05 | CDK4, CKS1B, CREBBP, CTNNB1, CYCS, FGF2, FGFR1, FIGF, IL8, NFKBIA, NRAS, PTCH1, VHL, WNT16 | AXIN2, BAX, CCDC6, COL4A2, CTBP2, E2F2, EGLN1, FGF13, GSK3B, HSP90AB1, ITGA2B, JUN, LAMC1, MAP2K2, PIK3CD, RAC2, RAC3, RALB, RARA, SLC2A1, SMO, STAT1, TPR, ZBTB16 |
| p53 signaling pathway | 2.65e-05 | TP53AIP1, CDK4, RRM2, BBC3 | PPM1D, CCNB1, TP53I3, CCNE1, SIAH1, BAX, EI24, GTSE1, STEAP3, CCND2, CCNB2, SFN | | 0.0002 | RFWD2, CDK4, CYCS | TP73, TSC2, SIAH1, EI24, BAX, STEAP3, SHISA5, CCNG1, CCNB1, CCND2 |
| Colorectal cancer | 0.0003 | CTNNB1, RAF1, AXIN1, PIK3R2, TCF7, MSH6 | TGFB1, AKT2, MSH2, PIK3CA, BAX, GSK3B, TCF7, TGFBR2 | | 0.0103 | CTNNB1, CYCS | JUN, RAC2, AXIN2, BAX, GSK3B, PIK3CD, RAC3 |
| [VEGF signaling pathway](http://www.kegg.jp/pathway/hsa04370+5296+5290+4893+208+4772+4775+9261+5894+56848+6300+8877+5600+63928+5829) | 0.0009 | NRAS, RAF1, PIK3R2, CHP2, MAPK12 | MAPKAPK2, NFATC3, PXN, AKT2, PIK3CA, SPHK1, MAPK11, SPHK2, NFATC1 | | ͞ | ͞ | |
| MAPK signaling pathway | 0.0070 | ATF4, CACNA2D3, CHP2, FGFR1, FLNA, MAP4K3, MAPK12, NRAS, PPM1B, RAF1 | AKT2, CACNA2D2, DUSP1, DUSP4, DUSP5, DUSP6, ELK1, FGF22, GNG12, MAP2K7, MAP3K6, MAPK11, MAPKAPK2, PRKACA, RAPGEF2, RPS6KA1, STK4, TGFB1, TGFBR2 | | 3.80e-06 | ATF4, CACNA1E, CACNB4, DUSP10, FGF2, FGFR1, LAMTOR3, MAP2K3, NRAS, PPM1B, RAP1B, RELB | CACNA1B, CACNA2D2, DDIT3, DUSP4, DUSP8, DUSP9, ELK1, FGF13, HSPA6, HSPB1, JUN, MAP2K2, MAP2K6, MAP2K7, MAP3K4, MAP3K6, NFATC2, PRKACA, RAC2, RAC3, RRAS, TAB2, TAOK2, ZAK |
| mTOR signaling pathway | 0.0088 | PIK3R2, RPS6KB2, DDIT4 | HIF1A, EIF4EBP1, RPS6KA1, AKT2, PIK3CA, ULK1 | | ͞ | ͞ | |
| Apotosis | 0.0297 | NFKBIA, IRAK2, PIK3R2, CHP2 | DFFA, IRAK1, AKT2, PIK3CA, BAX, TNFRSF10D, PRKACA | | 0.0113 | CASP7, NFKBIA, PRKAR1B, TNFSF10, CYCS | DFFA, IRAK1, BAX, PIK3CD, PRKACA, CFLAR |
| Stemness | | | | | | | |
| Wnt signaling pathway | 0.0003 | CTNNB1, AXIN1, NKD1, CHP2 | VANGL1, NKD2, CUL1, TCF7, WNT11, PPP2R5B, PSEN1, PRKACA, NFATC1, DAAM1, PPP2CA, NFATC3, SIAH1, PPP2R5C, GSK3B, LRP6, CCND2, PLCB2, CAMK2G | | 8.54e-05 | CSNK1E, CREBBP, CTNNB1, WNT16, CACYBP | VANGL1, NKD2, CUL1, NFATC2, CTBP2, LRP5, PSEN1, PRKACA, RAC3, DAAM1, JUN, RAC2, SIAH1, AXIN2, GSK3B, CCND2, CAMK2G |
| TGF-beta signaling pathway | 0.0110 | CDKN2B, ZFYVE16, ACVR1C, NOG, RPS6KB2 | TGFB1, PPP2CA, ID1, E2F5, TFDP1, CUL1, TGFBR2 | | 0.0381 | ZFYVE16, CREBBP, THBS2 | ID3, BMP7, E2F5, NOG, TFDP1, CUL1 |
| Hedgehog signaling pathway | 0.0130 | PTCH1, GAS1 | CSNK1G2, GLI3, RAB23, GSK3B, WNT11, PRKACA, CSNK1D | | 0.0156 | CSNK1E, PTCH1, WNT16 | BMP7, SMO, GSK3B, PRKACA, CSNK1D |
| Notch signaling pathway | 0.0365 | HES5, NCOR2 | HDAC1, NOTCH1, PSEN1, PSENEN, HDAC2 | | 0.0172 | CIR1, CREBBP | HES5, CTBP2, PSEN1, NCSTN, NUMB |
| Cell interaction | | | | | | | |
| Adherens junction | 5.11e-06 | SNAI1, ERBB2, CTNNA2, FGFR1, PTPRF, CTNNB1, CTNND1, MET, SNAI2 | LMO7, CTNNA1, TCF7, YES1, TGFBR2, SSX2IP, BAIAP2, MLLT4, PVRL2 | | 0.0014 | CREBBP, SORBS1, CTNNB1, FGFR1, SNAI2 | MLLT4, RAC2, INSR, ACP1, RAC3, SSX2IP, BAIAP2 |
| Tight junction | 0.0001 | NRAS, EPB41L1, CDK4, CTNNA2, MYH11, TJP2, CTNNB1 | CGN, CT͞͞͞͞͞NNA1, AKT2, MYH9, F11R, YES1, EPB41L2, MYL12A, PPP2CA, MLLT4, EPB41L3, CTTN, GNAI2, CLDN19, SPTAN1 | | 0.0157 | TJP2, ASH1L, NRAS, CTNNB1, CDK4, MAGI1 | MLLT4, CGN, EPB41L3, RRAS, LLGL2, ZAK, CLDN19, SPTAN1 |
| Regulation of actin cytoskeleton | 0.0013 | NRAS, RAF1, ENAH, FGFR1, ARPC3, GSN, PIK3R2, PIKFYVE | CYFIP1, GNG12, PXN, FGF22, ITGA5, MYH9, CYFIP2, BCAR1, ITGB5, MYL12A, BAIAP2, ARPC5, CSK, ITGA7, PIK3CA, PPP1CB, IQGAP3, CFL1, ARHGEF4 | | 0.0006 | NRAS, FGF2, ENAH, FGFR1, ARPC3, ITGA10 | RRAS, FGF13, PIP4K2C, BCAR1, ITGB5, MAP2K2, RAC3, ITGA2B, BAIAP2, VAV1, RAC2, PFN1, PPP1CB, PIK3CD, IQGAP3, ITGA9, GNA13, CFL1, LIMK2 |
| Cell adhesion molecules (CAMs) | 0.0040 | PVR, NRCAM, PTPRF, PTPRC, GLG1 | HLA-E, F11R, CADM1, SDC1, HLA-A, HLA-DMA, PVRL2, HLA-G, HLA-C, ICOSLG, CDH2, HLA-DOB, CLDN19 | | 0.0301 | PTPRC, CD276, CD22 | HLA-DMA, HLA-E, HLA-G, HLA-C, ITGA9, ICOSLG, CDH2, SDC1, HLA-A, CLDN19 |
| Focal adhesion | 0.0043 | RAF1, ERBB2, FLNA, CTNNB1, PIK3R2, MET | PXN, RAPGEF1, LAMC1, AKT2, ITGA5, COL4A5, ELK1, ILK, BCAR1, ITGB5, MYL12A, ITGA7, COL2A1, COL5A1, PIK3CA, PPP1CB, GSK3B, CCND2 | | 0.0001 | THBS2, RAP1B, FIGF, CTNNB1, ITGA10, CAV2 | COL4A2, LAMC1, ARHGAP5, ELK1, ILK, BCAR1, ITGB5, RAC3, ITGA2B, JUN, VAV1, PARVB, ZYX, RAC2, COL5A1, PPP1CB, GSK3B, PIK3CD, CCND2, ITGA9 |
| Gap junction | ͞ | ͞ | | | 0.0273 | NRAS, GUCY1B3, TUBB1 | GNAS, TUBB2A, TUBA1A, TUBB8, PRKACA, MAP2K2, CSNK1D |
| ECM-receptor interaction | ͞ | ͞ | | | 0.0394 | THBS2, ITGA10 | COL4A2, LAMC1, COL5A1, ITGA9, ITGB5, SDC1, ITGA2B |

**Table S2. Primer sequences used in RT-qPCR.**

| **Gene** | **Forward primer** | **Reverse primer** | **Size** |
| --- | --- | --- | --- |
| DDIT4 | CTGGACAGCAGCAACAGTG | ACACCCCATCCAGGTAAGC | 61 |
| MAGI1 | GAACAAGGACCTGCGACATTT | ACAGCATGGCGGTAAAGGTTA | 102 |
| PRPF19 | GAGCACCCATGTGTATCCCC | AGAGGCTGGTTGTTGATGGG | 104 |
| TUSC2 | GGAGACAATCGTCACCAAGAAC | TCACACCTCATAGAGGATCACAG | 139 |
| TBP | CCACTCACAGACTCTCACAAC | CTGCGGTACAATCCCAGAACT | 127 |
